# Anti-Diabetic Effects of Jiang Tang Xiao Ke Granule via PI3K/Akt Signalling Pathway in Type 2 Diabetes KKAy Mice

**DOI:** 10.1371/journal.pone.0168980

**Published:** 2017-01-03

**Authors:** Na Yu, Xin Fang, Dandan Zhao, Qianqian Mu, Jiacheng Zuo, Yue Ma, Yi Zhang, Fangfang Mo, Dongwei Zhang, Guangjian Jiang, Rui Wu, Sihua Gao

**Affiliations:** 1 Preclinical Medicine School, Beijing University of Chinese Medicine, Beijing, China; 2 Department of Endocrinology, Beijing University of Chinese Medicine Third Affiliated Hospital, Beijing, China; 3 Diabetes Research Center, Beijing University of Chinese Medicine, Beijing, China; 4 Department of Endocrinology, South Area of Guang’anmen Hospital, China Academy of Chinese Medical Sciences, Beijing, China; Tohoku University, JAPAN

## Abstract

Jiang Tang Xiao Ke (JTXK) granule, a Chinese herbal formula, has been used clinically to treat type 2 diabetes (T2DM) for decades. Our previous studies showed that JTXK granule exhibited anti-diabetic and anti-oxidative functions in experimental diabetic rats induced by a high fat diet and streptozotocin. However, the underlying mechanisms remain poorly understood. Herein, we aimed to investigate the therapeutic effect of JTXK granule on T2DM KKAy mice and the possible associations with skeletal muscle in the current study. Our results showed that JTXK granule significantly reduced food intake and body weight in T2DM KKAy mice. JTXK granule treatment also decreased the blood glucose and HbA1c levels and increased the insulin sensitivity in a time-dependent manner. Additionally, it ameliorated hyperlipidaemia and induced a lower free fatty acid level, displaying an effect on disorders of lipid metabolism. JTXK granule significantly increased the expression of insulin receptor substrate-1 (IRS-1), phosphoinositide 3-kinase (PI3K), protein kinase B (PKB/Akt) and glucose transporter 4 (Glut4) and decreased the expression of glycogen synthase kinase 3β (GSK3β). We concluded that JTXK granule is an effective drug for T2DM through regulating the PI3K/Akt signalling pathway in skeletal muscle.

## Introduction

Type 2 diabetes mellitus (T2DM) is becoming a worldwide health concern due to its high morbidity and mortality [1, 2]. Although many aspects of T2DM remain unclear, decreased insulin sensitivity and impaired insulin secretion are two generally recognized pathological changes inside the body during the progression of T2DM [3, 4]. Insulin resistance (IR) is a pathological state in which target tissues fail to respond normally to insulin, and tissues cannot easily absorb glucose from the bloodstream, thus contributing to the high level of blood glucose in the body [5]. Skeletal muscle is one of the most important peripheral tissue targets for insulin. Therefore, it is usually used as a significant therapeutic target in the battle against T2DM.

Glucose is metabolized in insulin sensitive tissues mainly through two pathways, phosphatidylinositol 3-kinase (PI3K) and 5’-AMP-activated kinase (AMPK) signal transduction pathways; the former is the classical one [6–8]. The major proteins in the PI3K signalling pathway, such as PI3K, protein kinase B (PKB/Akt) and glucose transporter type 4 (Glut4), play an important role in insulin transduction [9]. After insulin connects with the receptor in the membrane of target cells, the signal cascade is triggered. The activated insulin receptor promotes tyrosine phosphorylation of insulin receptor substrate-1 (IRS-1), leading to PI3K and Akt activation as well as downstream lipid and glucose metabolism [10]. Increasing evidence has revealed that the expression and phosphorylation of Akt are associated with T2DM [11]. Glut4 and GSK3β, which are downstream of PI3K, are the pivotal proteins in controlling glucose uptake and storage and glycogen metabolism [12, 13]. Therefore, the drugs that could regulate the above proteins are promising in the treatment of T2DM.

Existing antidiabetic agents are often associated with side effects or insufficiency [14]. Therefore, there is a need for new drugs for T2DM prevention and treatment. Currently, multiple approaches have been applied and attempted, among which traditional Chinese medicine has played an inevitable role and achieved satisfactory results [15, 16]. Jiangtang Xiaoke (JTXK) granule is one of a series of Chinese prescriptions invented by Dr. Sihua Gao, under the guidance of the study of “kidney, liver and spleen in the dialectical treatment of T2DM”. The above study has won the second prize of National Scientific and Technological Progress Awards for its advantages and universal applicability [17]. JTXK granule is superior to other Chinese medicine because it is a good reflection of combination of Chinese medicine and western medicine as it not only accords with Chinese medicine theory, but also bases on modern pathophysiological and pharmacological research. JTXK granule contains ten herbs, including Radix rehmanniae (DiHuang), Fructuscorni (ShanYuRou), Radix salviae miltiorrhizae (DanShen), Rhizoma coptidis (HuangLian), Pueraria (Gegen), etc. Previous studies have demonstrated that some of the above active ingredients may act on PI3K signalling pathway [18, 19]. JTXK granule has been clinically demonstrated to be effective in controlling high glucose and to relieve the symptoms of T2DM patients since the 1980s. Previously, we found that JTXK granule could decrease both serum glucose and lipid levels in experimental diabetic rats induced by a high-fat diet and streptozotocin through protecting against damage to the islets and anti-oxidative action [20]. However, the molecular mechanisms of JTXK granule are still to be explored in KKAy mice, a wild type 2 diabetic rodent model, which exhibits marked obesity, glucose intolerance, severe IR, dyslipidaemia, and hypertension [21]. Hence, we investigated the effect of JTXK granule on T2DM KKAy mice and its association with the PI3K/Akt signalling pathway in muscular tissues in the present study.

## Materials and Methods

### Animals

A total of 60 eight-week-old KKAy male mice and 10 age-matched C57BL/6J male mice were purchased from Beijing HFK Bioscience Co. Ltd. (Beijing, China). The study was conducted in strict accordance with the recommendations in the Guide for the Care and Use of Laboratory Animals from the Association for the Committee for Animal Experiments of the National Centre. The protocol was approved by the Ethics Committee of Beijing University of Chinese Medicine. The mice were housed individually in a temperature (23 ± 2°C) and humidity (55 ± 10%) controlled environment with a 12 h light/dark cycle with free access to food and water.

### Drugs

JTXK granule was produced according to the method reported in previous literature [22]. Briefly, the raw herbs of JTXK granule (Radix rehmanniae (DiHuang), Fructuscorni (ShanYuRou), Radix salviae miltiorrhizae (DanShen), and Rhizoma coptidis (HuangLian), Pueraria (Gegen), etc.) were purchased from Beijing Tong Ren Tang Pharmacy (Beijing, China), and Professor Zexin Ma (TCM museum at Beijing University of Chinese Medicine) verified their authenticity.

For the preparation of JTXK granule, some herbs were boiled in twelve volumes of distilled water for 1 h. The pooled aqueous extract was filtered through a gauze cloth and then evaporated by heating until the relative density reached 1.15. The rest of the herbs were extracted three times with twelve volumes of 60% (v/v, in H2O) ethanol under reflux. JTXK granule was obtained by mixing the pooled aqueous and ethanoic extracts with a yield of 20% (w/w) (i.e., 5 g of herbs for every 1 g of extract). The main components of the JTXK granule were detected by a HPLC fingerprint system (S1 Appendix). The prepared JTXK granule was stored at 4°C until use.

### Study design

The KKAy mice were fed with a high-fat diet, and the C57BL/6J mice were fed with a standard commercial diet, provided by Beijing HFK Bioscience Co. Ltd. (Beijing, China). Blood glucose was checked with a blood glucometer (Mishawaka, USA) once a week to monitor the condition after inducing diabetes. Those KKAy mice whose random blood glucose level was higher than 13.9 mmol/L were judged as T2DM KKAy mice and included in this study. Fifty T2DM KKAy mice were then randomly divided into either the vehicle treated group (Con), the pioglitazone-treated group (Piog), or the JTXK-treated group (JTXK high, JTXK medium, JTXK low). Age-matched C57BL/6J mice were also dosed with a vehicle as healthy non-diabetic control animals (Nor). Pioglitazone was given at a dose of 4.5 mg/kg/d. JTXK granule was delivered by oral gavage into the animal’s stomach at a dosage of 7, 3.5, or 1.75 g/kg/d for 10 weeks. The non-diabetic control and diabetic control groups received an equal volume of vehicle (saline). Food intake, body weight and fasting blood glucose levels (with glucose meters) were monitored weekly. An oral glucose tolerance test (OGTT) was conducted at week 5 and week 10. After 10 weeks of the intervention, all mice were sacrificed by cervical dislocation under anaesthesia with pentobarbital sodium after fasting for 12 h. Plasma samples were collected and stored for biochemical analysis. The skeletal muscle tissues were immediately removed and stored at −80°C for subsequent RT-PCR and western blotting analysis.

### Reagent

Pioglitazone hydrochloride was purchased from Jiangsu Hengrui Medicine Co., Ltd. (H20040267, Lianyungang, China). First Strand cDNA Synthesis Kits were bought from Promega Corporation (Cat #: #1622, Madison, USA). The SYBR Premix was provided by Applied Biosystems (Cat #: 4367659, Foster, USA). The primers were designed and synthesized by Sangon Biotech (Shanghai, China). Antibodies to GAPDH, PI3Kp85, Akt, p-Akt (ser473), GSK3β were purchased from Abcam, Inc. (Cat #: ab8245, ab189403, ab8805, ab18206 and ab32391, Cambridge, UK). The antibodies to IRS-1 and Glut4 were purchased from Cell Signaling Technology (Cat #: #3194 and #2213, Beverly, USA). Anti-Rabbit IgG-HRP and Anti-mouse IgG-HRP were from Santa Cruz (Cat #: sc-2030 and sc-200, California, USA).

### Biochemical analysis

Serum blood glucose, insulin and haemoglobin Alc (HbAlc) levels were determined at the end of the study with automatic biochemistry analyzer. The index of the homeostasis model assessment of insulin resistance (HOMA) was calculated as fasting plasma glucose [mmol/L] × fasting plasma insulin [mU/L]/22.5. The insulin sensitive index was calculated as 1/[FBG (mmol/l)×FINS (μU/ml)]. Serum levels of high density lipoprotein cholesterol (HDL-C), low density lipoprotein cholesterol (LDL-C), total cholesterol (TC) and triglycerides (TG) were measured using commercial enzyme-linked immunosorbent assay (ELISA) kits (Nanjing, China) according to the manufacturer’s instructions. Blood plasma free fatty acid (FFA) content was measured using an enzymatic assay (Clinimate NEFA, Tokyo, Japan).

### Oral Glucose Tolerance Test (OGTT)

Animals were fasted overnight, and then, oral glucose load was administered at a dose of 2 g/kg. Glucose levels were measured from the tail vein before and 30, 60, and 120 min after glucose administration. The glucose tolerance was evaluated by calculating the area under the curve (AUC).

### Real-time PCR

Total RNA was extracted from soleus muscle biopsies using TRIZOL reagent (Invitrogen Life Technologies, USA) according to the manufacturer’s instructions. Then, the purity was confirmed, and the concentration of each sample was measured using a spectrophotometer. Then, total isolated RNA was reverse-transcribed to synthesize cDNA using the commercial kit. The cDNA was then used as the template to amplify the target genes using real-time PCR (Bio-rad, USA) in a 20-μL total volume of 2 μL of cDNA, 10 μL of SYBR Premix, 1.0 μL of forward primer, 1.0 μL of reverse primer, and 8 μL dH_2_O. Quantitative real-time PCR was performed using the following protocol as recommended by the manufacturer (ABI 7500, USA): 1 cycle at 50°C for 2 min and 95°C for 10 min, followed by 40 cycles at 95°C for 15 s and 60°C for 1 min. The sequences of the primers are summarized in S1 Table. All PCR data were normalized to beta-actin gene expression.

### Western blotting analysis

Skeletal muscles were lysed in RIPA buffer supplemented with complete protease inhibitor cocktail (Roche, USA), and the protein concentration was determined using a BCA Protein Assay Kit (Thermo Scientific, USA). A total of 50 μg of denatured protein sample was separated by 10% of the SDS-PAGE gel and transferred onto a PVDF membrane. After being blocked with 5% skim milk in TBST (Tris-buffered saline containing 0.1% Tween 20) for 1.5 h at room temperature, the membranes were then incubated overnight at 4°C with a primary antibody (1:1000 dilution), followed by incubation with the corresponding HRP-labelled secondary antibody (1:5000) at room temperature for 1.5 h on the second day. The immune complex was detected using the ECL kit according to standard procedures (Thermo Fisher Scientific Inc., China). Protein bands were visualized using autoradiography, and the intensities were analysed using Quantity One (Bio-Rad Laboratories, USA). GAPDH was used as the loading control.

### Statistical analysis

SPSS software, version 17.0, was used for the statistical analyses. All data are shown as the means ± standard error (M ± SEM). One-way ANOVA was used for multiple group comparisons, followed by LSD or SNK test for the comparison between two groups. P values <0.05 were considered significant.

## Results

### JTXK granule decreased the body weight and food intake in T2DM KKAy mice

**Fig 1:**
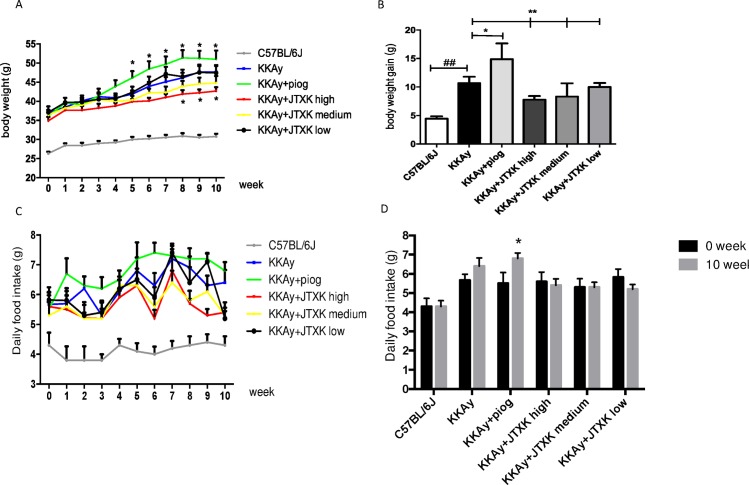
JTXK granule decreased the body weight and food intake in T2DM KKAy mice. (A) Body weight change of the mice during treatment in each group. (B) Comparisons of the body weight gain among between groups. (C) Daily food intake of the mice during treatment in each group. (D) Comparisons of the daily food intake before and after the experiment in each group. Data are expressed as the mean ± SE of ten mice in each group. *P<0.05, **P<0.01 vs. control group (T2DM KKAy mice). ## P<0.01 vs. non-diabetic control group (C57BL/6J mice). ΔP<0.05 vs. daily food intake before experiment.

The body weight and food intake of the mice are shown in Fig 1A and 1B. The body weight of the mice gradually increased during the experiment, particularly the mice in the control group. The average body weight gain reached 10.67 g in the control group. Compared with the body weight gain (4.45 g) of C57BL/6J mice, the difference was significant (P<0.01). This finding suggested that T2DM KKAy mice in the current study were obese, which is a major characteristic of T2DM. At the end of the study, the average body gains of the piog group and the JTXK high, medium and low groups were 14.89 g, 7.78 g, 8.33 g and 10.33 g, respectively. Compared with the mice in the control group, treatment with pioglitazone improved the body weight gain of the diabetic mice, whereas JTXK granule inhibited it (P<0.01). The amount of food intake by the diabetic mice was significantly greater than the non-diabetic control mice (Fig 1C). In comparing the food intake before and after the experiment, we found that the food intake of the mice in the control group and the piog group significantly increased (P<0.01), but the food intake in the three JTXK granule treated groups did not significantly change (P>0.05) (Fig 1D). The results indicated that JTXK granule may reduce body weight by adjusting the food intake of the diabetic mice.

### JTXK granule reduced the blood glucose level and haemoglobin A1c

**Fig 2:**
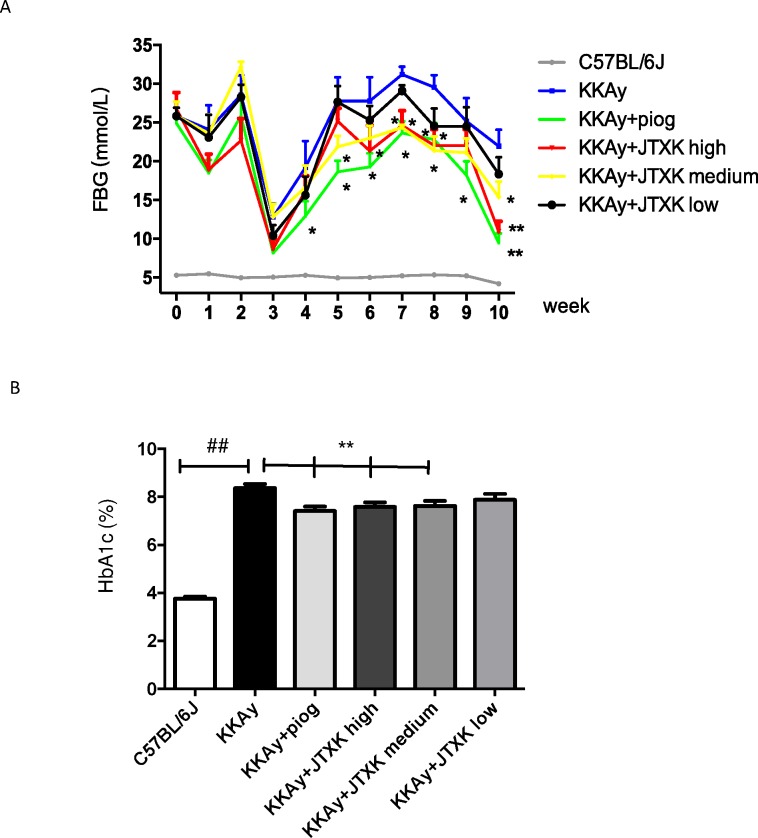
**JTXK granule reduced the blood glucose level (A) and haemoglobin A1c (B) in T2DM KKAy mice.** Data are expressed as the mean ± SE of ten mice in each group. *P<0.05, **P<0.01 vs. control group (T2DM KKAy mice). ## P<0.01 vs. non-diabetic control group (C57BL/6J mice).

The blood glucose level of T2DM KKAy mice fluctuated during the study. Specifically, the glucose level dropped from the second week to the fifth week (the mice were 11 to 14 weeks old) and then increased gradually. Compared with the control group, pioglitazone significantly reduced the glucose level as expected. The administration of JTXK granule reduced the blood glucose level to some extent, as well. The blood glucose levels in the high and medium JTXK dose groups were lower compared with the control group, particularly at the end of the experiment (P<0.01) (Fig 2A). As shown in Fig 2B, the HbA1c level of mice in the control group was more than two times that of the C57BL/6J mice. Compared with the control group, the HbA1c levels in the piog and high, medium and low JTXK dose groups were decreased by 0.95%, 0.78%, 0.73%, and 0.45%. The difference between the first three groups and the control group was significant (P<0.01).

### JTXK granule decreased the plasma insulin concentration and increased insulin sensitivity in T2DM KKAy mice

**Fig 3:**
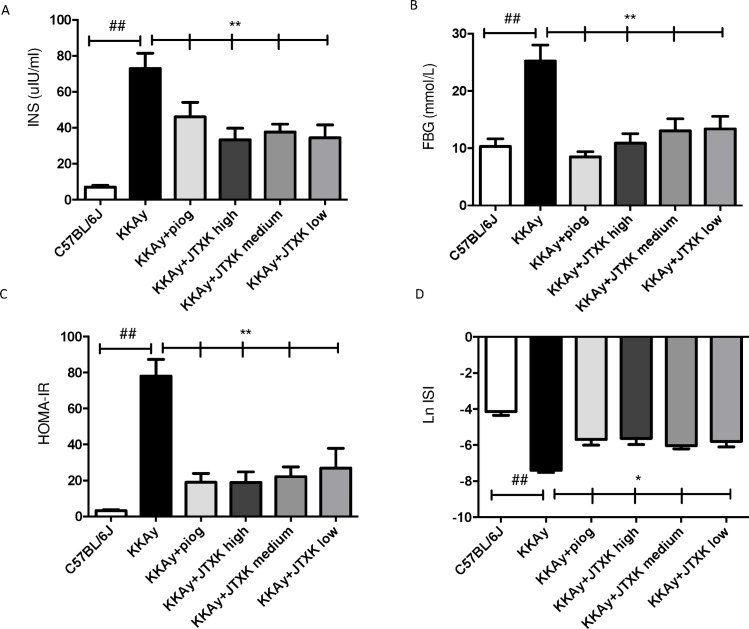
JTXK granule decreased the plasma insulin concentration, and improved insulin sensitivity in T2DM KKAy mice. (A) Serum insulin concentration was reduced by JTXK granule at the end of the treatment. (B) Serum fasting glucose levels of the mice in each group. (C) HOMA-IR of the mice in each group. (D) ISI of the mice in each group. Data are expressed as the mean ± SE. *P<0.05, **P<0.01 vs. control group (T2DM KKAy mice). ## P<0.01 vs. non-diabetic control group (C57BL/6J mice). HOMA-IR = FBG (mmol/l)×FINS (μU/ml)/22.5; ISI = 1/[FBG (mmol/l)×FINS (μU/ml)].

The HOMA-IR and ISI were calculated using the formula mentioned above in the study design. As shown in Fig 3A, the plasma insulin concentration of the diabetic mice was significantly higher than that of the C57BL/6J mice and was reduced with pioglitazone and JTXK granule treatment (P<0.01). Fig 3B states the plasma glucose level after ten weeks of the treatment. According to the results of HOMA-IR and ISI (Fig 3C and 3D), the insulin sensitivity was lower in the diabetic mice compared with the C57BL/6J mice. Administration of pioglitazone and JTXK granule for ten weeks significantly enhanced insulin sensitivity and relieved the IR in the T2DM KKAy mice.

### Effects of JTXK granule on OGTT in T2DM KKAy mice

**Fig 4:**
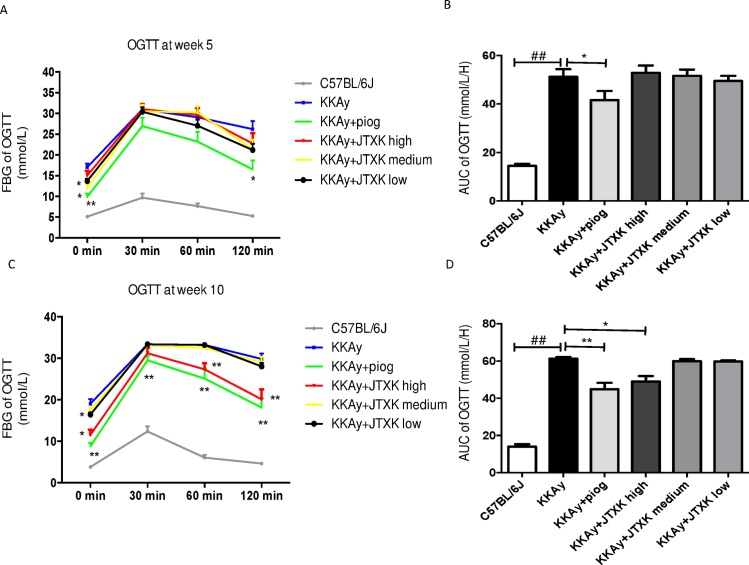
JTXK granule improved results of oral glucose tolerance test (OGTT) in T2DM KKAy mice. A and C showed results of OGTT in each group at the fifth and the tenth week. B and D showed area under the curve (AUC) of the OGTT among groups at the fifth and the tenth weeks. Data are expressed as the mean ± SE of ten mice in each group. *P<0.05, **P<0.01 vs. control group (T2DM KKAy mice). ## P<0.01 vs. non-diabetic control group (C57BL/6J mice).

An OGTT was conducted at weeks 5 and 10, and the results are shown in Fig 4. Compared with the C57BL/6J mice, the blood glucose concentration and AUC were higher in the diabetic mice. After 5 weeks of treatment, the basal glucose concentrations in the medium and low JTXK dose groups and the piog group were significantly lower than that of the mice in the control group. However, JTXK granule did not reduce the blood glucose levels or AUC significantly after the glucose load (Fig 4A and 4B). After 10 weeks of drug supplementation, the piog group showed effective glucose removal throughout the experiments after the glucose load (P<0.01). Similarly, high dose JTXK granule also reduced the AUC and the glucose levels at 60 min and 120 min. However, a similar action was not observed in the medium and low JTXK dose groups, demonstrating that oral glucose tolerance was not affected by medium and low doses of JTXK granule (Fig 4C and 4D).

### JTXK granule decreased serum lipid profile and FFA level

**Fig 5:**
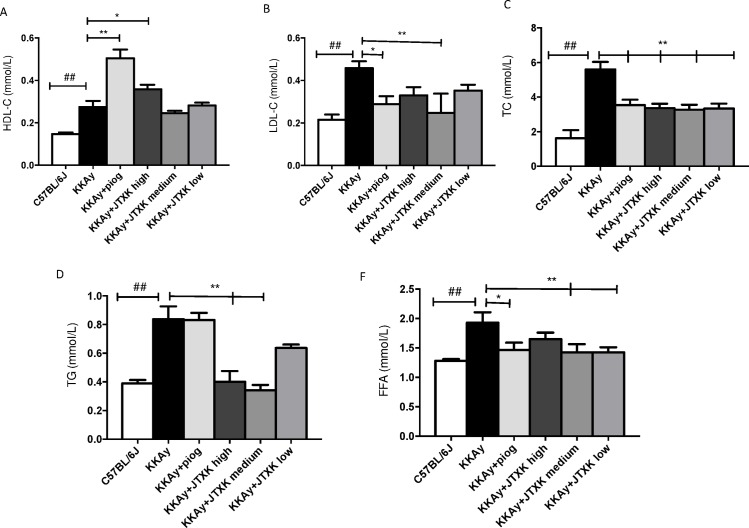
JTXK granule improved serum lipid levels and decreased FFA level in T2DM KKAy mice. HDL-C, high density lipoprotein; LDL-C, low density lipoprotein; TC, total cholesterol; TG, triglycerides; FFA, free fatty acid. Data are expressed as the mean ± SE of ten mice in each group. *P<0.05, **P<0.01 vs. control group (T2DM KKAy mice). ## P<0.01 vs. non-diabetic control group (C57BL/6J mice).

As shown in Fig 5, the levels of LDL-C, TG, TC and FFA were significantly increased, and the level of HDL-C was markedly decreased in the T2DM KKAy mice compared with the non-diabetic C57BL/6J mice (P<0.01). A high dose (7 g/kg) of JTXK granule and pioglitazone showed a similar effect on increasing HDL-C and decreasing LDL-C and TC; HDL-C increased by 30.75% (P<0.05), and LDL-C and TC decreased by 45.88% and 39.77%, respectively. However, medium (3.5 g/kg) and low doses (1.75 g/kg) of JTXK granule did not show a significant influence on HDL-C and LDL-C levels. Similarly, TG levels in the high dose (7 g/kg) and medium dose (3.5 g/kg) JTXK granule groups reduced by 52.10% and 59.27%. Additionally, the medium and low doses of JTXK granule resulted in a significant reduction (25.99% and 26.09%, respectively) in the serum FFA level. The results suggested that JTXK granule, particularly at a high dose, had a beneficial effect on regulating serum lipid levels and FFA level.

### Effects of JTXK granule on mRNA expression in the PI3K/Akt signalling pathway in skeletal muscle

**Fig 6:**
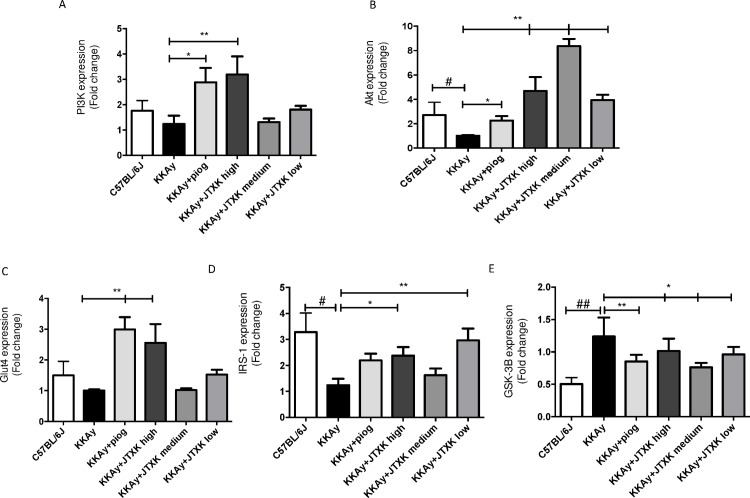
JTXK granule altered mRNA expression in the PI3K/Akt signalling pathway in skeletal muscle. IRS-1, insulin receptor substrate-1; PI3K, phosphatidylinositol 3-kinase; Akt, serine/threonine-protein kinase; p-Akt, phosphorylated Akt; Glut4, glucose transporter 4; GSK3β, Glycogen synthase kinase 3 β. Data are expressed as the mean ± SE of six mice in each group. *P<0.05, **P<0.01 vs. control group (T2DM KKAy mice).

As shown in Fig 6, the mRNA expression of PI3K, Akt, Glut4 and IRS-1 in the piog group was significantly higher and the GSK3β expression was significantly lower compared to those in the control group (P<0.05). The various doses of JTXK granule also increased the mRNA expression of PI3K, Akt, Glut4 and IRS-1 and reduced GSK3β expression. Specifically, the mRNA expression of PI3K in the high JTXK dose group was 3 times higher compared with that in the control group (Fig 6A). The mRNA expression of Akt was up-regulated by supplementation with various doses of JTXK granule (Fig 6B), whereas the mRNA expression of GSK3β was down-regulated (Fig 6E). Moreover, the high dose of JTXK increased the Glut4 and IRS-1 by more than two times (Fig 6C and 6D).

### Effects of JTXK granule on relevant protein expression in the PI3K/Akt signalling pathway in skeletal muscle

**Fig 7:**
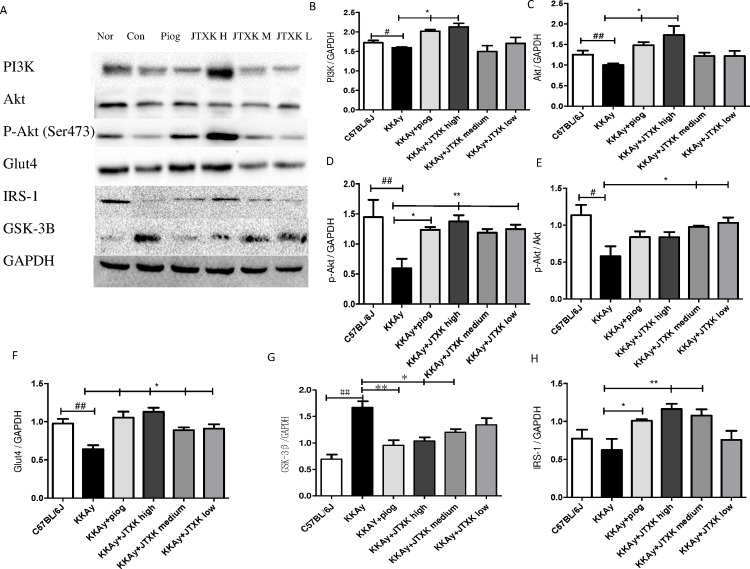
JTXK granule influenced relevant protein expression in the PI3K/Akt signalling pathway in skeletal muscle. IRS-1, insulin receptor substrate-1; PI3K, phosphatidylinositol 3-kinase; Akt, serine/threonine-protein kinase; p-Akt, phosphorylated Akt; Glut4, glucose transporter 4; GSK3β, Glycogen synthase kinase 3β. Data are expressed as the mean ± SE of six mice in each group. *P<0.05, **P<0.01 vs. control group (T2DM KKAy mice). ## P<0.01 vs. non-diabetic control group (C57BL/6J mice).

The expression of the proteins PI3K, Akt, p-Akt, p-Akt/Akt, Glut4 and IRS were also significantly increased at various doses of JTXK granule (Fig 7), and this finding was consistent with the mRNA expression in the various groups. Additionally, the protein expression of GSK3β in the JTXK granule groups decreased. The results indicate that JTXK granule treatment may active the PI3K signalling pathway, which helps control glycaemia in mice.

## Discussion

TCM has gradually been accepted as an effective method to prevent the development of diabetes [23, 24]. However, the complicated composition and unclear pharmacological targets limit its universal international application [25]. In the current study, JTXK granule, an important herbal formula for the treatment of diabetes, is constructed according to the TCM theory [17]. JTXK granule clinically exhibited effective regulation in controlling blood glucose levels and the associated complications. Our previous animal experiments have also proved its hypoglycaemic and antioxidant effects [20]. Additionally, the stable preparation process of JTXK granule has been determined after long-term researches. The herbal drugs of JTXK granule were verified using fingerprinting techniques, and the main ingredients were also determined, including berberine, paeonol, salvia acid, puerarin, coptisine and etc. (shown in sup.1). Our group has already conducted research on the functions and working mechanisms of the components, such as berberine, curcumin, ginsenoside and Corni Fructus [26–29]. In addition, the results from other researchers have also demonstrated anti-diabetic effect of some active ingredients of JTXK granule [30].

IR is a widespread phenomenon in obesity and T2DM. Referred to as a decline in insulin bioavailability, IR is the main pathological change that occurs in T2DM [31] and is also an important part in the development of many other diseases such as obesity, hypertension, and hyperlipidaemia [32]. Therefore, developing effective drugs for controlling IR has become a popular research objective. In the present study, pioglitazone, a common insulin sensitizer, was chosen as the positive control drug to confirm the effect of JTXK granule on IR. Consistent with our previous researches, JTXK granule demonstrated a favourable effect on lowering the blood glucose level and HbA1c in T2DM KKAy mice. It may also control the food intake and body weight of diabetic mice. As reported, obesity is a significant risk of diabetes and a fertile ground for IR. In most obese individuals, increased food intake is to be blamed. A study showed that approximately 70% of diabetic patients had a food addiction, and their body mass index was higher compared to that of people who did not meet the food addiction criterion [33, 34]. Hence, we deduced that relieving food addiction and controlling food intake contributed to the pharmacological functions of JTXK granule in T2DM KKAy mice. Additionally, JTXK granule improved insulin sensitivity based on the results of the OGTT and HOMA-IR index. There was not a dose-dependent effect, even though different doses of JTXK granule were administered to the mice. The interaction among the complex ingredients of JTXK granule may be the reason for the above results. If the dose of the entire formula is increased, the positive actions of some ingredients may be offset by the others. Moreover, the effect of JTXK granule on reducing body weight and improving OGTT showed a time-dependent manner.

Lipid metabolism disturbance is commonly found in patients with T2DM [35]. A disorder of the lipid metabolism induces the excessive production of FFA and endocrine factors, which subsequently decreases the biological activity of insulin [36]. Fasting plasma FFA levels correlate inversely with insulin sensitivity through its action on glucose oxidation. Consequently, it is considered a predictor of the development of T2DM. JTXK granule was found to have a certain effect on regulating lipid metabolism. Specifically, it can reduce LDL-C, TC and TG levels and increase HDL-C levels. The decreased level of FFA observed in the results was thought to be one of the key working targets of JTXK granule on regulating IR.

Skeletal muscle is a major tissue responsible for insulin-dependent glucose utilization [37]. Several factors have been implicated as the major defects responsible for IR in muscle tissues during the progression of T2DM, among which FFA was taken as a key point in recent studies [38, 39]. It has been generally recognized that a lack of exercise is an important cause of T2DM, and proper movement is a meaningful strategy for treating T2DM.

Insulin exerts its physiological functions through the post receptor cascade after binding to the insulin receptor on the surface of the target cell membranes. The dominant regulation occurs via the IRS/PI3K signal transduction pathway [40]. PI3K is a key protein involved in glucose metabolism and the insulin physiologic effect. Activated PI3K increases phosphorylated Akt, which further activates the downstream signalling molecules such as FOXO, GSK3β, and Glut4 [12, 13, 41]. Thus, the PI3K signalling pathway mediates glucose uptake and intracellular glycogen synthesis in skeletal muscle tissues and glucose dysplasia and glucose output in the liver [42]. Oxidative stress in the body can lead to the occurrence of IR. In IR, the PI3K signalling pathway is inhibited [43, 44]. Consequently, the transportation of glucose to the plasma membrane is hindered. We previously found that JTXK granule had an anti-oxidant effect. In accordance with the literature, the results of our study showed decreased expression of the protein in the PI3K signalling pathway in the skeletal muscle of T2DM KKAy mice. JTXK granule was found to significantly suppress the increase in the gene and protein expression levels of IRS-1, PI3K, Akt and Glut4 and decreased the up-regulation of the expression of GSK3β in T2DM KKAy mice. The results suggest that JTXK granule influences glucose uptake and utilization and glycogen synthesis in the skeletal muscle by adjusting the actions of the PI3K signalling pathway.

## Conclusions

Our findings showed that T2DM KKAy mice demonstrated the typical characteristics of IR, and JTXK granule treatment reduced the blood glucose level and lipid profiles and relieved IR in diabetic mice through regulating the PI3K/Akt signalling pathway in skeletal muscles. The results suggest that JTXK granule seems to offer potential as a plant extract that is useful for alleviating metabolic syndrome.

## Supporting Information

S1 Appendix Fingerprint Chromatogram of JTXK Granule. (PDF)

S2 Appendix Histological Pictures of Muscle Tissues. (PDF)

S1 Table List of Primer Sequences for RT-PCR. (PDF)
