# Supplementary material for: Anti-Diabetic Effects of Jiang Tang Xiao Ke Granule via PI3K/Akt Signalling Pathway in Type 2 Diabetes KKAy Mice
Source: PLoS One. 2017 Jan 3;12(1):e0168980. doi: 10.1371/journal.pone.0168980 (PMC5207690; doi:10.1371/journal.pone.0168980)
Supplement: S1 Appendix — (PDF) [file pone.0168980.s001.pdf]

A.

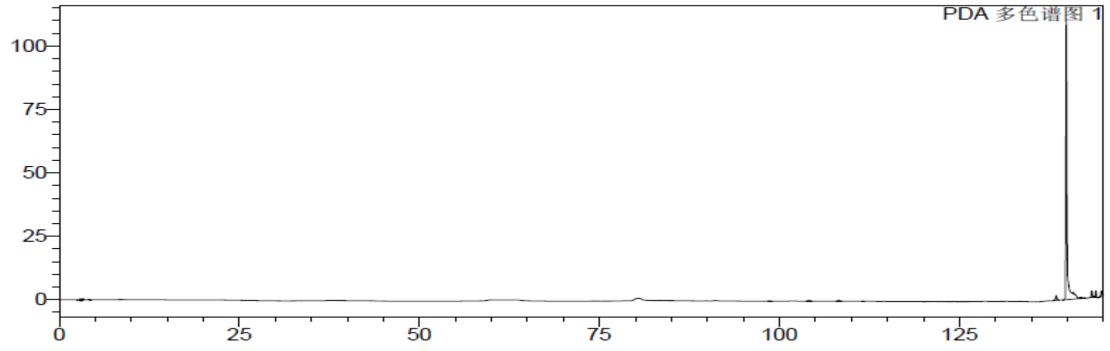

PDA 多色谱图 1
100
75
50
25
0
0
25
50
75
100
125

B.

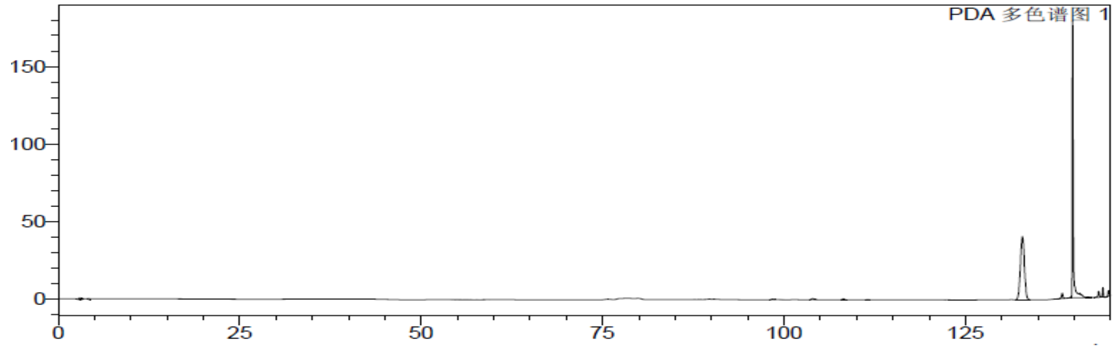

PDA 多色谱图 1
150
100
50
0
0
25
50
75
100
125

C.

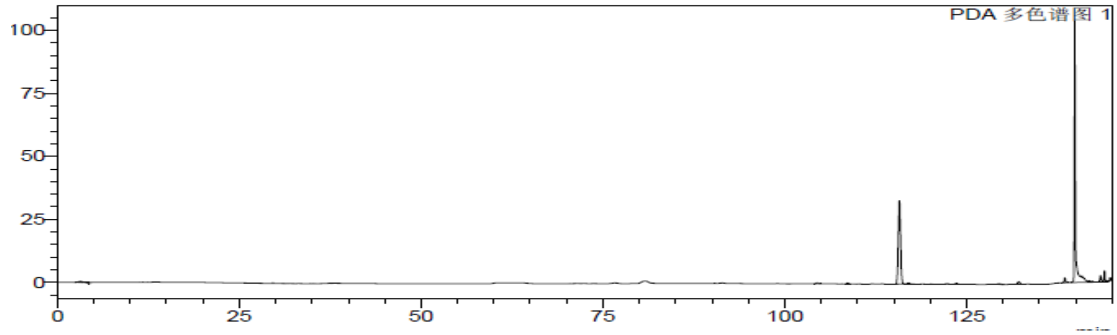

PDA 多色谱图 1
100
75
50
25
0
0
25
50
75
100
125

D.

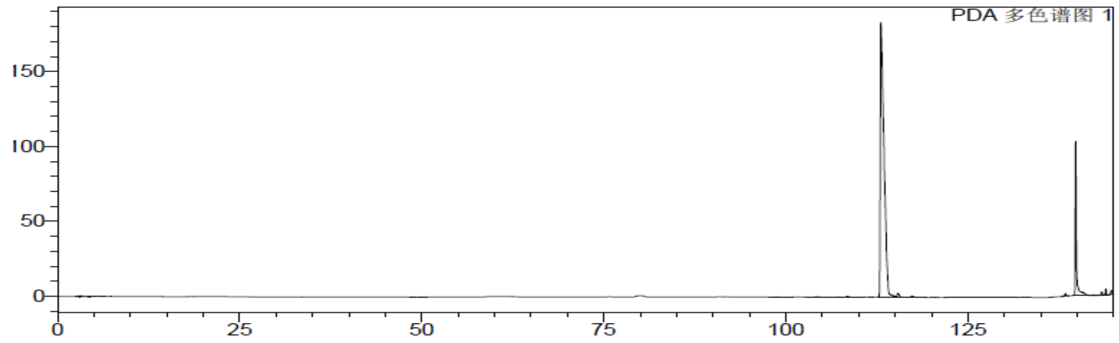

PDA 多色谱图 1
150
100
50
0
0
25
50
75
100
125

E.

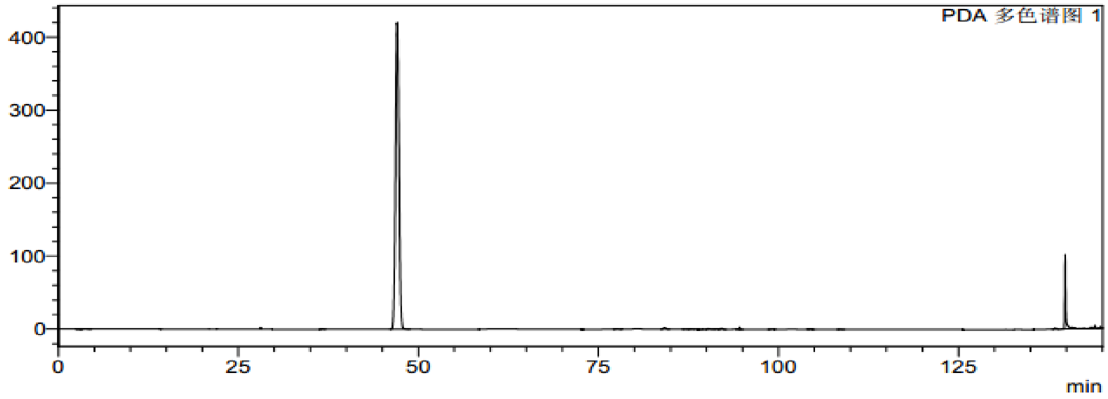
PDA 多色谱图 1
400
300
200
100
0
0
25
50
75
100
125
min

F.

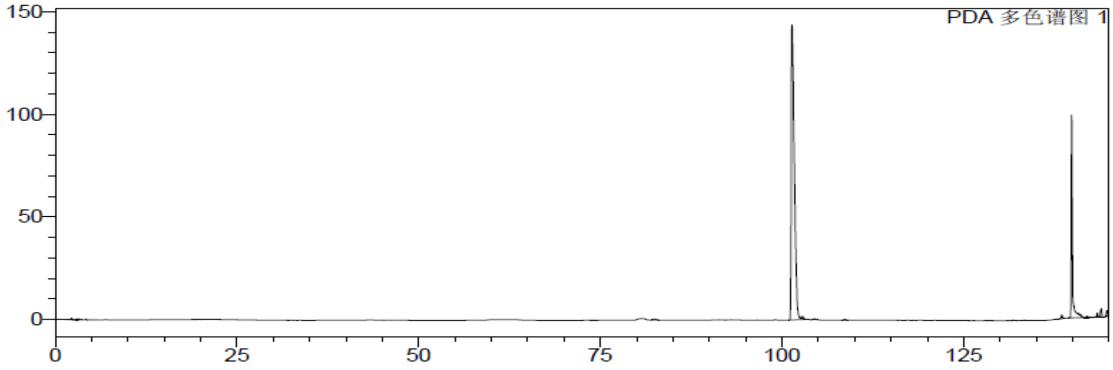
PDA 多色谱图 1
150
100
50
0
0
25
50
75
100
125

G.

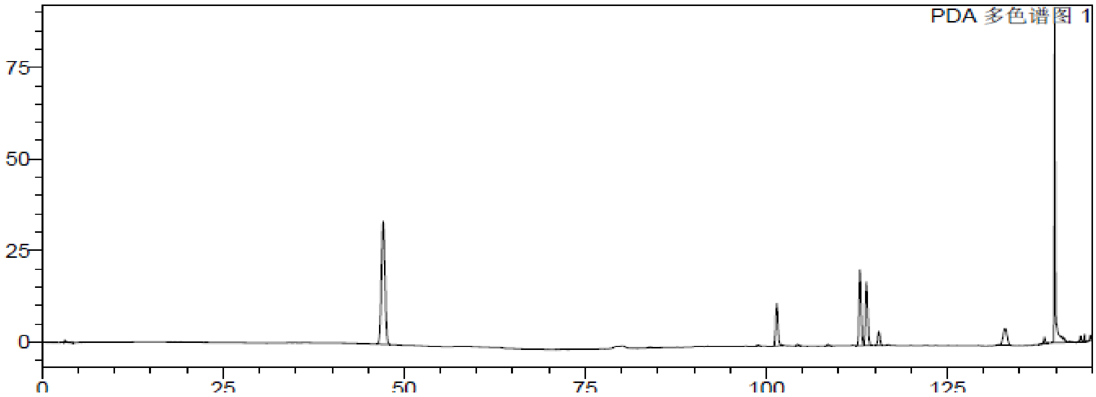
PDA 多色谱图 1
75
50
25
0
0
25
50
75
100
125

H.

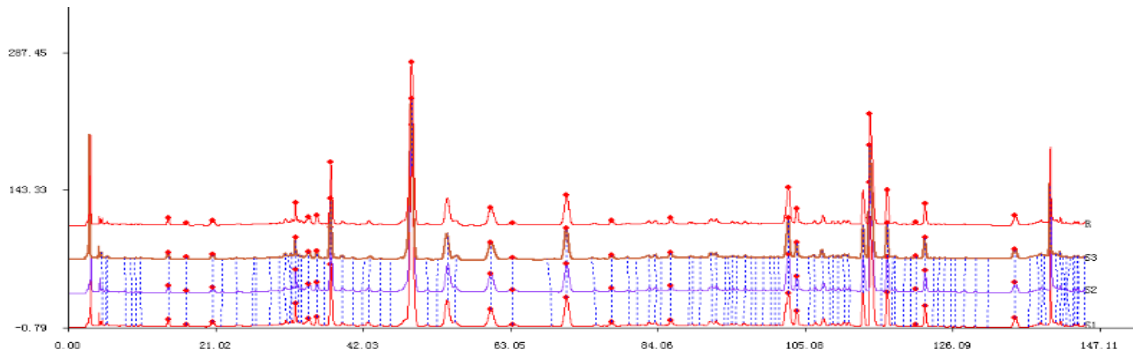

I.

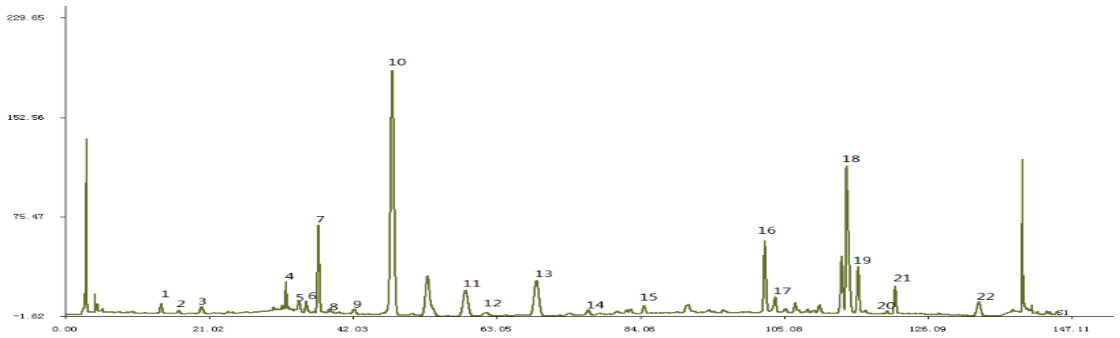

Appendix 1. Fingerprint Chromatogram of TJXK Granule

(A) Chromatogram of blank methyl alcohol. (B) Reference chromatogram of paeonol. (C) Reference chromatogram of salvia acid B. (D) Reference chromatogram of berberine. (E) Reference chromatogram of puerarin. (F) Reference chromatogram of coptisine. (G) Chromatogram of mixture reference substance. (H) Fingerprint chromatogram of TJXK granule was established by using sample chromatography of 3 different production batches. (I) Twenty-two common fingerprint peaks were found in fingerprint chromatogram of TJXK granule.
