# Supplementary figures and images for: Anti-Diabetic Effects of Jiang Tang Xiao Ke Granule via PI3K/Akt Signalling Pathway in Type 2 Diabetes KKAy Mice
Source: PLoS One. 2017 Jan 3;12(1):e0168980. doi: 10.1371/journal.pone.0168980 (PMC5207690; doi:10.1371/journal.pone.0168980)

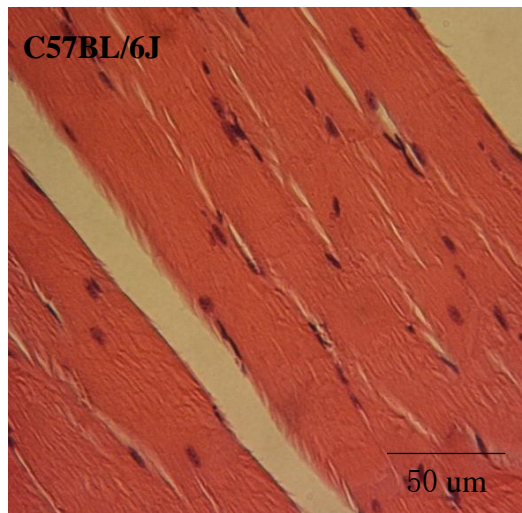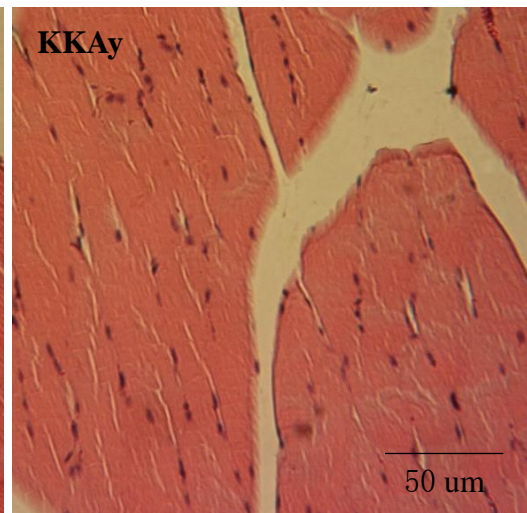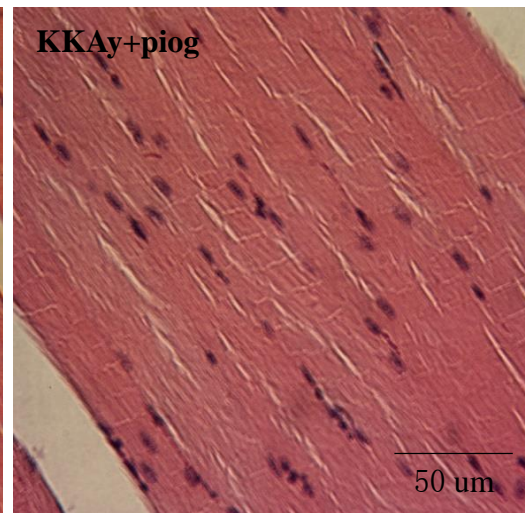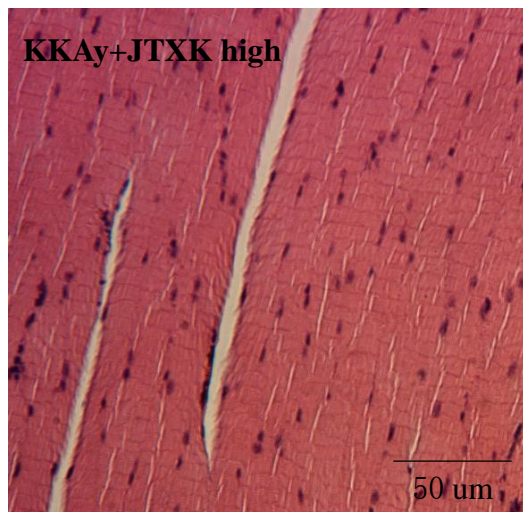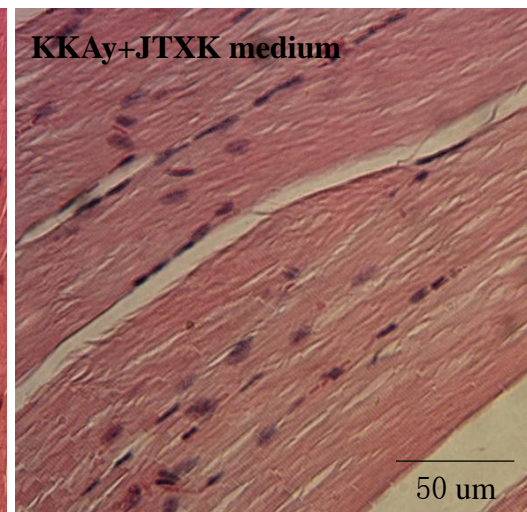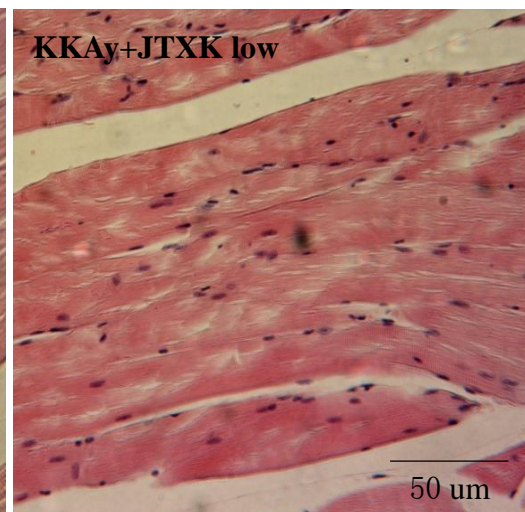

Supplement: S2 Appendix — (PDF) [file pone.0168980.s002.pdf]
