# Supplementary material for: Anti-Diabetic Effects of Jiang Tang Xiao Ke Granule via PI3K/Akt Signalling Pathway in Type 2 Diabetes KKAy Mice
Source: PLoS One. 2017 Jan 3;12(1):e0168980. doi: 10.1371/journal.pone.0168980 (PMC5207690; doi:10.1371/journal.pone.0168980)
Supplement: S1 Table — (PDF) [file pone.0168980.s003.pdf]

Table 1 List of Primer Sequences for RT-PCR.

Gene	primer	sequences
IRS-1	Forward (5’→3’)	CAGGCACCATCTCAACAATC
	Reverse (3’→5’)	GTTTCCCACCCACCATACTG
Pik3r1	Forward (5’→3’)	CCTTGGAGAGGTTTGACCATTA
	Reverse (3’→5’)	CATCCCAGCTATGCTGTATCTATC
Akt1	Forward (5’→3’)	GGCAGGATGTGTATGAGAAGAA
	Reverse (3’→5’)	GTGATCATCTGAGCTGTGAACT
GSK-3β	Forward (5’→3’)	GCCAATGCAGAGGTCCTAAA
	Reverse (3’→5’)	CGAAAGGGAAGAGAAGGTAACA
Glut4	Forward (5’→3’)	CCCACAGAAAGTGATTGAACAG
	Reverse (3’→5’)	AGAGAGCCCAAAGGGTAGTGAG
β-actin	Forward (5’→3’)	CACCCGCGAGTACAACCTTC
	Reverse (3’→5’)	CCCATACCCACCATCACACC
